# Inequitable care delivery toward COVID-19 positive people of color and people with disabilities

**DOI:** 10.1371/journal.pgph.0001499

**Published:** 2023-04-19

**Authors:** Danbi Lee, Paula M. Kett, Selina A. Mohammed, Bianca K. Frogner, Janice Sabin

**Affiliations:** 1 Department of Rehabilitation Medicine and Disability Studies Program, University of Washington, Seattle, WA, Untied States of America; 2 Center for Health Workforce Studies, Department of Family Medicine, University of Washington, Seattle, WA, Untied States of America; 3 School of Nursing and Health Studies, University of Washington Bothell, Bothell, WA, Untied States of America; 4 Department of Biomedical Informatics and Medical Education, University of Washington, Seattle, WA, Untied States of America; Southern Cross University, AUSTRALIA

## Abstract

This study aimed to explore provider observations of inequitable care delivery towards COVID-19 positive patients who are Black, Indigenous, and Other People of Color (BIPOC) and/or have disabilities and to identify ways the health workforce may be contributing to and compounding inequitable care. We conducted semi-structured interviews between April and November 2021 with frontline healthcare providers from Washington, Florida, Illinois, and New York. Using thematic analysis, major themes related to discriminatory treatment included decreased care, delayed care, and fewer options for care. Healthcare providers’ bias and stigma, organizational bias, lack of resources, fear of transmission, and burnout were mentioned as drivers for discriminatory treatment. COVID-19 related health system policies such as visitor restrictions and telehealth follow-ups inadvertently resulted in discriminatory practices towards BIPOC patients and patients with disabilities. As patients experience lower quality healthcare during the pandemic, COVID-19-related restrictions and policies compounded existing inequitable care for these populations.

## Introduction

It is well-documented that Black, Indigenous, and People of Color (BIPOC) as well as people with disabilities experience discrimination and bias in the health care they receive [1–4]. Actions and attitudes related to this include stereotyping; delayed, less or lower quality of care; failure to provide interpreters; negative assumptions about adherence to care resulting in different treatment options; and less patient-centered communication [2, 5–10]. For example, studies show that implicit bias among providers is associated with longer wait and appointment times for Black patients as compared to Whites [7] and Black patients are more likely to report poor interpersonal experiences [8] with less patient-centered interaction and more “clinician-dominated visit dialogue” [6].

Studies examining provider bias concerning people with disabilities have found provider misconceptions regarding supports needed to access care or fill out forms; reports of providers ignoring or minimizing patient concerns; as well as assumptions that certain services were unnecessary, such as an accessible parking pass [11–13]. One study found discriminatory practice against people with disabilities such that more than half of physicians reported not being likely to welcome people with disabilities into their practice [11]. While no specific reason was given as to why, this same study did state that a majority of physicians reported lower levels of confidence in being able to provide the same quality of care for patients with disabilities as for their other patients, which may be an underlying reason for this discriminatory practice [11].

Such biases and discriminatory actions in care, in addition to the long history of structural racism inherent in the U.S. health system, underlie current inequities in health outcomes affecting BIPOC populations and people with disabilities [2, 3, 14, 15]. Among BIPOC populations, discriminatory treatment results in delayed or missed diagnoses; experiences of racism in care also contribute to reduced treatment adherence and delayed preventive care which are connected to worse health outcomes [6, 14, 16, 17]. Further, structural and historical inequities as well as discrimination restricting access and opportunity have resulted in higher rates of chronic disease in BIPOC populations [15, 18]. Likewise, people with disabilities experience disparities in access to the social determinants of health and preventive services; have more instances of delayed care, are more likely to rate their health as fair or poor; and have disproportionately higher rates of injuries and chronic disease [1, 19–21].

These existing inequities in care and health outcomes have been further exposed and exacerbated by the COVID-19 pandemic. The disproportionate rates of COVID-19 morbidities and mortality among BIPOC populations and people with disabilities have been widely reported [15, 18, 22]. These populations experience greater risk due to social and systemic inequities; experts have also highlighted the ways that discrimination and bias in healthcare further increases susceptibility to complications and mortality from COVID-19 [23, 24].

Provider burnout and emotional distress due to the pandemic [25, 26] could exacerbate COVID-19 inequities, as studies have found an association between such workforce outcomes and racial bias in care [27, 28]. However, few studies have examined such inequities in healthcare during the COVID-19 pandemic; those that do exist focus on the patient perspective or the healthcare worker’s own experience of discrimination [29, 30]. A deeper understanding how bias among providers may be negatively affecting treatment decisions or quality of care related to COVID-19 is needed [31, 32]. In this study, we examine frontline providers’ observations of inequitable care delivery towards COVID-19 positive patients who are BIPOC and/or have disabilities and health workforce related drivers and facilitators contributing to and compounding inequitable care. We chose to explore this from the provider perspective as this offers additional insight related to the influence of the work environment as well as to situations of which the patient may be unaware.

## Methods

The study used a qualitative design with individual semi-structured interviews to capture healthcare providers’ perspectives on equitable care delivery toward COVID-19 patients who are BIPOC and/or have disabilities. Interviews took place between April and November 2021.

### Ethics statement

The University of Washington Institutional review Board determined the study qualifies for exempt status (Approval# STUDY00011837). Participants provided a verbal consent.

### Recruitment

Frontline providers were recruited using convenience and snowball sampling. We included any healthcare providers who provided care to COVID-19 patients during the pandemic in Washington, Florida, Illinois, and New York. These four states were selected with the goal of regional diversity as well as their varying state COVID-19 management and policies. We selected to recruit from major hospitals and Federally Qualified Health Centers located in counties that had the highest number of cases in the four states, which were primarily urban areas. Flyers and email invites were sent to Chief Medical Officers, Chief Nursing Officers, and individual providers in critical care, emergency care, pulmonary, infectious disease, and rehabilitation, using their publicly available email addresses. Invites were also sent to the authors’ personal contacts who could distribute the information to clinical units providing care for COVID-19 patients. Follow-up e-mails were sent up to three times to maximize the response. To ensure transferability, recruitment continued until we reached saturation on themes, where interviews primarily produced previously discovered data.

### Data collection

Thirty-minute individual interviews were conducted by two to three research team members via Zoom. The interview guide included questions regarding equity in applying crisis standards of care protocols towards COVID-19 patients who are BIPOC and/or have disabilities, equity in treatments among those patients, and perceptions around stigma related to COVID-19. Questions were developed by a team with expertise in health and healthcare equity, implicit bias, health workforce, nursing, rehabilitation, and disability studies research and based on known disparities in care reported in the literature. Cultural appropriateness was validated with input from four project advisory board members who are advocates representing the BIPOC and disability communities. Participants received a copy of the interview guide prior to their interview. The same set of questions was consistently used across interviewers. Interviews were recorded with permission and transcribed verbatim; in addition, one team member took notes during each interview.

### Data analysis

Using Dedoose software, four authors engaged in thematic analysis of the interviews. The team ensured credibility and dependability of the study, by following the analysis steps suggested by Braun and Clarke [33]. The first five transcripts were each coded by the four authors, allowing for discussion to refine codes and reach consensus as a group. The team also referred to interview notes when developing the codes. After finalizing the codebook, the four authors coded the subsequent transcripts in pairs where they engaged in an iterative comparison, dialogue, and consensus process, ensuring dependability of findings [33]. The team then collectively reviewed the codes and grouped them into major themes and subthemes. To ensure confirmability and acknowledge the potential bias that the research team members have, coming from academic backgrounds (i.e., faculty and research scientist) and identifying as cisgender, non-BIPOC and/or nondisabled, we engaged in reflexivity by frequently debriefing and inviting the advisory board to review our analyses and provide feedback. Findings for this paper focused on participants’ observations of inequitable care.

## Results

**Fig 1:**
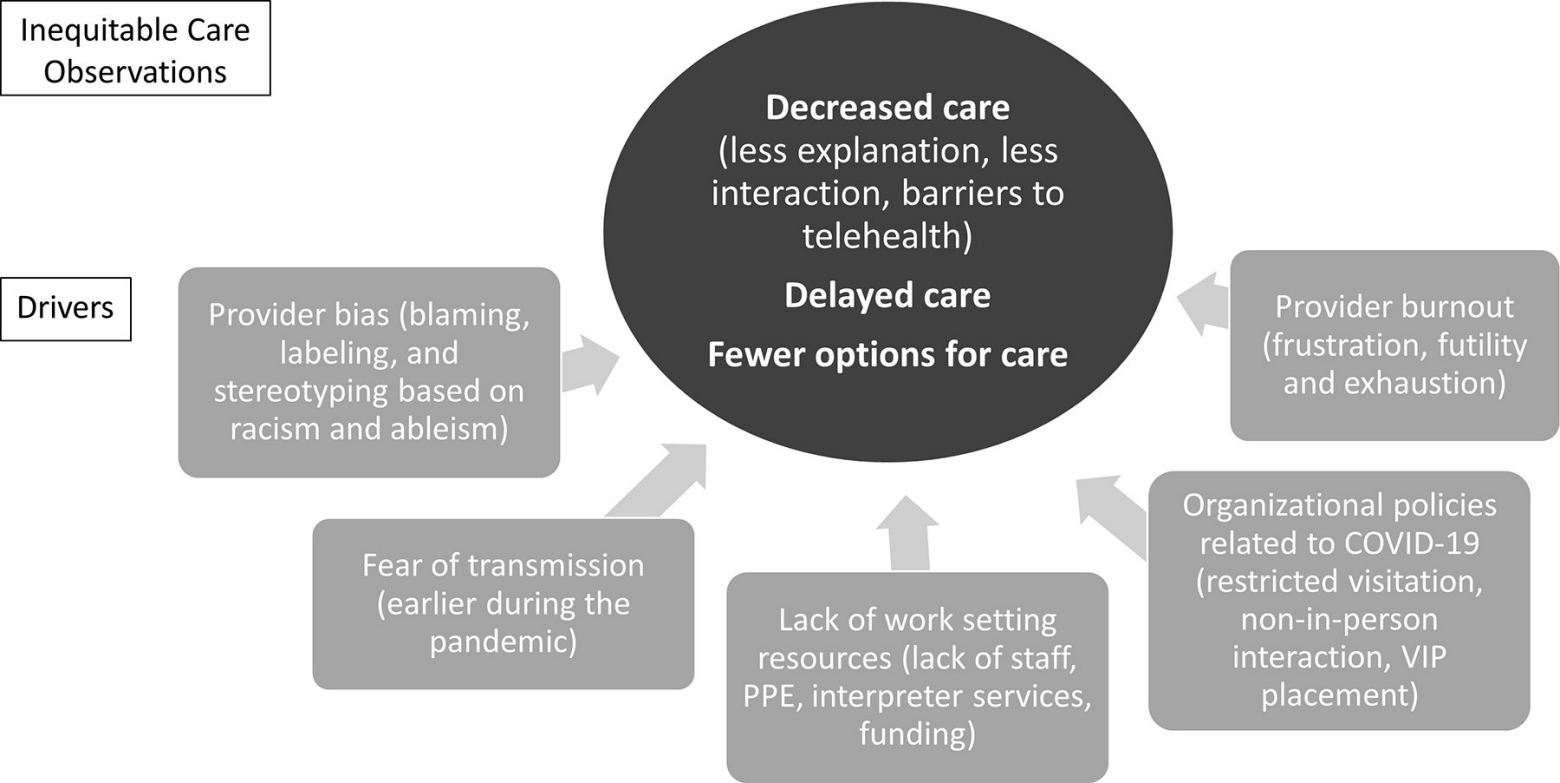
Model illustrating major themes.

A total of 19 pandemic frontline healthcare providers participated in the study, including nine physicians, five nurses, three rehabilitation professionals, and two nurse practitioners. The settings in which participants practiced included Emergency Medicine (n = 4), Critical Care (n = 6), Rehabilitation medicine (n = 3), Pediatric Primary Care (n = 2), Pediatric Emergency Care (n = 1), and Pediatric Critical Care (n = 2) across 9 major urban hospitals, 2 FHQCs, and Veteran’s Administration. The majority of participants were female (n = 12) and White (n = 11). Participants were from one of the four targeted states–Washington (n = 5), New York (n = 5), Florida (n = 5), and Illinois (n = 4). The majority of participants reported observations of inequitable care in the form of *decreased care*, *delayed care*, and *different options for care*, often due to the perceived additional needs these patient groups might require based on language barriers, cultural differences, lower socioeconomic status, and physical or mental impairments. Within these observations, participants described a number of drivers underlying inequitable care, including bias connected to *racism and ableism*, *patient mistrust*, *provider-patient disconnect*, *organizational policies*, *lack of work setting resources*, *and provider burnout* (Fig 1). Two participants reported they did not observe inequitable care in their setting.

### Inequitable care observations

#### Decreased care

Participants reported observations of decreased care in terms of less explanation, less interaction, and barriers in access to telehealth services among BIPOC patients and patients with disabilities. A majority of participants noted how lack of accommodations for communication resulted in less explanation of care. This was frequently observed among patients with limited English proficiency (LEP) because access to interpreter services was limited or not sought. One participant highlighted the complexities of caring for COVID -19 patient with LEP:

*“It does create difficulties, especially as the care gets more technical and more complicated, to bring the information to the patient, because you’re so sick and you’re supposed to listen to the doctor or the nurse and to understand complex issues. And also then, you ask the person, well, what would you like to have done? How does he or she make that decision? […] it is difficult to take care of patients who have a language barrier on any given day, but I think COVID just adds to that dynamic just with everything else that’s going on.”*

Lack of accommodation for communication was also observed with deaf patients or patients with cognitive disabilities. Participants highlighted ways that information such as discharge instructions did not get clearly communicated due to lack of accommodation. One participant noted the ways it impacted care especially when provider biases were added to the equation:

*…when there’s not that direct transmission of information, there’s things that are lost, there’s things that are misinterpreted…Even something as simple as [with] COVID-19, you’re supposed to prone all the time whether you’re independent, whether you’re on a ventilator, you’re supposed to prone. And that message I feel like is often just brushed away because they’re like ‘that’s not that crucial to them.’”*

Participants further shared concerns regarding their observations of providers choosing alternative ways to communicate (e.g., informal interpretation by staff, writing out for deaf patients) out of convenience or due to lack of resources. Participants noted that *“[T]hings were not necessarily explained to the fullest capability"* with such alternatives and that “*messages weren’t coming across clearly*.”

Several other participants noted the impact of communication barriers on patients’ healthcare experiences. One participant shared their observation with a patient with LEP: *"[The patient] didn’t understand because he didn’t speak English…[it] didn’t impact his medical care*, *but it impacted him personally for sure…it did play a role in his care*.*"* For a deaf patient with LEP, the staff did not offer the necessary support: “*he was just lost and confused with what was happening half the time because whenever he was…having a lot of trouble*, *people weren’t stopping to explain it very well to him*.*"* While these patients may have received the same medical treatment, their care experiences and mental health during their stay would be negatively impacted.

Decreased care was observed also in the form of less interaction with patients. In some settings where physicians communicated with COVID-19 patients only via phone, patients requiring accommodations for communication were deprived from routine physician visits or visits from specialists. This included people with LEP and people with cognitive disabilities such as individuals with dementia or Down syndrome.

*"During the height of COVID, the majority of our physicians weren’t even going in the rooms, they were observing outside the room or making phone calls into the room with these folks who some of them, they couldn’t even understand English; or B, don’t even understand the concept of like picking up a phone and talking it on it anymore because their dementia is so bad or their cognitive impairment is so bad. So, absolutely, the time that those patients were communicated with, and things were explained, […] was limited."*

In addition, participants observed healthcare providers explicitly expressing ableism and racism of BIPOC patients and patients with disabilities, evident also in less interaction with or differential treatment of these groups. Participants reported patients with disabilities or chronic conditions being avoided or treated unequally by healthcare providers primarily due to their internalized ableism. Participants noted how ableism overtly affected *“individual level decisions”* related to the care of people with disabilities and at times resulted in negligent care: *"If someone comes in more chronically ill*, *or more disabled*, *the same efforts are not always put in*.*"* Several participants specifically highlighted the ways those with cognitive impairments were particularly vulnerable.

*"They just didn’t want to give him [patient with Down syndrome] the time in his care that I felt like he needed and he deserved to make sure they were examining him properly, make sure that they weren’t missing anything with him. … it [was] kind of a neglective care like, ‘I’m not going to really pay much attention, he doesn’t really get it, he’s not paying attention to what I’m doing,’ was kind of the overall feeling I got in rounds which I did not love."*

*“I heard multiple staff members say that they were paying more attention to other patients than that patient [who was at risk of dying at any time] because they didn’t believe her to be likely to survive. And she was no different than any other patient other than the fact that she had trisomy 21."*

Similarly, racism and race-based biases impacted the care of BIPOC patients. Healthcare providers’ biases, such as assuming higher pain tolerance in the Hispanic/Latinx community, resulted in different care: *"They [the Hispanic/Latinx community] have higher pain tolerances’ is something I heard over and over again*.*" "…I feel like people of color with their symptoms…sometimes they’re taken less seriously*, *there’s less compassion*.*”* It was noted by several participants, however, that decreased care based on racism was held in check by providers’ fear of punishment and risk of loss of license.

Finally, decreased care due to issues with accessing telehealth services was frequently cited as a limited option for people with LEP and those with disabilities. One participant emphasized:

*“There’s limitations in terms of access to technology. If you are visually impaired, can you log onto a computer or a phone to start a video visit with the physician? Then some people were not sort of—did not have enough technology literacy to complete that process.”*

Another participant spoke to the limitations of telehealth from a language perspective, noting

*“Most of our discussions surrounding telehealth and access to care [have been around] knowing that there are limitations from the technology end, from the language barrier, and just being able to read the instructions in English which is normally how they’ve been sent, of how to log onto a video visit, but we have a lot of patients who speak Spanish, who speak Polish…”*

#### Delayed care

In addition to receiving decreased care, participants also reported instances of BIPOC and people with disabilities experiencing delays in care. These delays were often attributed to the busy environment and additional resources required (e.g., staff, devices, interpretation). When discussing how language barriers affect delays in care, one participant stated:

*[the] system is already set up to not automatically care for people who don’t speak English as their first language…[patients] get put back into the room with less information than…a patient who spoke English as their first language. And that leads to a barrier of care…it takes longer to see that patient and for them to get the attention that they might get if they didn’t have that language barrier.*

Particularly, one person shared how a patient who will require more time due to interpreter needs would be seen later if they were deemed to be medically stable:

*Basically if you’re going to use an interpreter, your visit’s going to take 2X as long because everything has to be said twice… when you see a patient, [if] you already know they’re stable, because the triage nurse has seen them and you can watch their vitals. […]. So, I think if you’re busy, […] you know it’s going to take you more time, so you hesitate a little bit more before you add that patient to your plate.”*

Another participant expounded upon the same phenomena with an example from the Emergency Department of which patient assignment system was set up to allow avoidance:

*I think the main thing that happens with the care decision is just it takes an ED [Emergency Department] to notice people because in the ED we choose our patients. We like watch the board and then we pick a patient. And you watch those patients sit there, nobody picks them for a while, the providers.*

In other instances, participants emphasized the conscious and active nature of delayed care when working with patients with disabilities, particularly those who have physical disabilities or are obese: "*with Down Syndrome*, *those kids tend to be very floppy*, *some of them…you need a lot of hands*.*"* For such patients, one participant said that *people are often very thoughtful about when they’re going to go into the room because they know it will be a big ordeal…because it’s more difficult sometimes*, *it gets done at a later time*."

The delayed care in ancillary services (these include physical therapy, occupational therapy, or respiratory therapy) that patients with disabilities received then often resulted in decreased care, as one participant stated:

*“I think if they [ancillary service providers] know it’s going to be something that’s going to take a lot of time, they’ll either do it really early or really late. And if it gets done really late or if it gets scheduled really late, and sometimes other things take some time, then that person gets missed for the day.”*

Participants shared how such delays in care contributed to patients feeling like *“there’s nobody taking care of them”* and overall experience care that was “*less than optimal*.*”*

*Fewer options for care*. Often times, patients who were BIPOC or who had physical or mental disabilities were given fewer options to care. When discussing this occurrence among people of color, socioeconomic status was often conflated with race. As one provider stated:

*“I saw a lot of instances where options were not being presented to people of color. And they would fault those social economic factors as, like, rationale. But at the end of the day that is very unethical in my opinion.”*

In response to a question regarding differences in care for COVID-19 positive people of color or those with disabilities, one participant shared this observation where less long-term treatment options were provided:

*“Another instance that I witnessed, […] this patient was intubated. And normally what gets offered is a tracheostomy. And then what gets offered is an LTACH [Long Term Acute Care Hospital] facility if this patient is no longer going to be able to come off the ventilator. That was not offered to this patient. That was not an option. What was pushed instead was comfort care and compassionate extubation. That was very eye opening because that’s not how that normally goes. And so essentially, this woman’s life has ended, not on my shift but later that week instead of having options of what it is that she would have wanted.”*

### Drivers

#### Provider bias

Provider bias, particularly bias related to racism and ableism, was a major driver to differences in care described above. Such bias was evident in observations of certain provider attitudes and overheard comments. Participants recounted hearing conversations, often described as *“side-conversations”* occurring before and after care, which included blaming language, *“trash talking*,” and occurrences of labeling, generalizing, stereotyping, or making assumptions based on race, ethnicity, and/or presence of impairments. The intersectionality of these characteristics compounded negative attitudes toward patients. These conversations included expressions of frustration or irritation related to patients’ specific needs or cultural differences with patients as well as lack of compassion for a patient’s situation. For example, several participants shared providers’ statements about undocumented patients as *“eating up resources”*, characterizing them as a burden or as someone who is taking advantage of the system–*“he’s being waited on hand and foot*, *who wouldn’t want that*.*”*

Similar comments related to a patient’s socioeconomic status (SES) were also described by participants, particularly regarding a patient’s sanitation or barriers to discharge due to lack of resources or accommodations. One participant’s observation illustrates how the intersectionality of SES and racial bias results in discrimination:

*“[One] instance I can think of was a Black […] woman, middle age, [and] she was homeless. [I didn’t see] overt racism, […] more microaggressions. She had lice, along with all the other COVID problems, so nursing are heavily involved in lice care for patients. [There were] just a lot of comments around her hair texture and difficulty with hair management and not so compassionate in terms of how they were approaching it. […] it was just a lot of like comments around ‘well, if her hair wasn’t so ratty’ and, ‘if her hair was just properly kept’, ‘If it wasn’t so unkempt,’ and just sort of things like that.”*

Expression of irritation was also shown in observations of referring to expressions of grief among Hispanic/Latinx populations as “*Hispanic panic”* or describing Black/African American patients as *“aggressive” or “crazy”* due to communication style differences. Some participants also expressed frustration with differences in cultural and religious beliefs that impacted care decisions. For example, one participant shared the cultural clash with Hispanic caregivers who disagreed with provider’s recommendations of care:

*“…family is very important and taking care of your family is what they do. I’ll see patients who are elderly who live with their children. That is just something that culture seems to really do. And so focusing on [I’m] taking them home, being with me, I’m going to protect them, I’m going to take care of them is part of it.”*

Participants described additional evidence of racial bias, such as categorizing based on a patient’s race or *“automatically grouping a person [from a different racial group] […] into ‘they’*.*”* This resulted in negative patient-oriented comments providers would make based on assumptions about the larger racial or ethnic population the patient was a part of. One participant highlighted ways these assumptions were applied to insinuate that Hispanic/Latinx patients were at fault for contracting COVID-19:

*“At the nursing station alone, it would be like, "Well, they cohabitate.’ ‘Well, they aren’t wearing their masks.’ ‘They aren’t staying home.’ […] This person comes in […] and the saddest part about all of this is that, they’re young […] and they are otherwise healthy, and all of a sudden, within two minutes, this nurse, or this NP, or this doctor knows exactly how they got here. […] And it’s, like, ‘you have no clue’.”*

Finally, bias related to ableism was evident in “*subjective assessment[s] of quality of life”* that providers were observed to make with people with disabilities or chronic conditions, reinforcing beliefs that their lives are worth less or that being *“less likely to survive”* justifies less attentive care. Providers were also observed to blame disabilities or pre-morbid conditions for worse health outcomes as the patients’ fault.

*“…I have heard statements that, basically, if that person was not as obese as he is, that this would most likely not be this severe right now. Or it wouldn’t be so many other components that were failing and would not create additional problems.”*

Due to the *“mortality rate of this condition in older adults”* providers would also express frustration if an elderly person with pre-existing medical conditions took the last bed, illustrating how ageism intersects with ableism: *“you could also sort of hear people say ’this person’s 85*, *they have advanced dementia*. *You know*, *do they get my last ICU bed*?*’”*

As the pandemic wore on, participants shared bias against patients who were unvaccinated, which in some cases the majority of unvaccinated were people of color. Participants shared that “*there is less compassion”* and “*significant bias*” toward this population, compounding existing concerns for racial bias. They noted that while they did not believe this changed the care that was provided, it did change *“the way that the care was delivered*,” highlighting that the *“conversations*, *not necessarily to the patient*, *but within the clinical arena*, *[were] different based on that*.*”* One participant shared this observation:

*“We had a patient who was African American […] Didn’t believe COVID existed, didn’t believe in the vaccine. He, I think, was treated unfairly. He kept saying this doesn’t exist, stop telling me I have COVID and they would just yell at him basically ‘you have COVID, you have to believe it.’ And he just refused to believe it. And so I do feel like that does lessen the compassion. He ended up coding four times and unfortunately, he did not make it. And so, I don’t know, I wasn’t there for the code and so I don’t know how his attitude was portrayed to the code team, but they talked about it multiple times like he’s a non-compliant patient. He doesn’t listen."*

#### Fear of transmission (earlier during the pandemic)

Participants shared the fear that existed among providers, particularly early on in the pandemic when there was less understanding of the virus, which also contributed to differences in care, particularly issues with less care or avoidance of the patient all together. This fear resulted in providers declining to use translation services because they “*were afraid to pick up the phone”* or care being done faster than normal to reduce exposure. One participant described their observations related to this:

*“There’s the putting on all the PPE and going to see a patient and then just basically like trying to do what you need to do and then get out of there. I think that was really the—that was the recurring pattern I think for everyone out of fear of the unknown, concern for our well-being and just being afraid of basically getting potentially exposed.”*

#### Organizational policies related to COVID-19

Hospital COVID-19 policies such as restricted family visitation, speaking with providers via electronic devices, and VIP patient placement (patients with certain connections or status would be placed preferentially on established units as opposed to temporary COVID-19 units where there was less privacy and fewer resources) contributed to issues of inequitable care. The inability to have family or caregiver support in the hospital amplified challenges related to interpretation, because as one participant noted, “*95% of the time [a patient’s] family translates for them*.*”* Outside of interpretation, patients with LEP often relied on family members in understanding their care, and without this support one participant said “*you just have this person laying there confused and doesn’t know what’s happening*.*"*

Family members would also often serve as advocates if the patient was not familiar with the healthcare system or required physical, mental, and emotional support or to ensure high quality care: *“if a person of color has an advocate…I feel like you see a little bit of change in behavior from a medical provider because there is that additional accountability*.*”* The need for having an advocate was also insinuated in a comment on differential power dynamics in the provider-patient relationship which directly influences how comfortable patients feel communicating with providers:

*“Patients don’t say what they think to the providers, and instead only share them with their family…It just makes me question how do they perceive us, when we walk in in our gear and equipment, and especially when the doctor visitations are with all these doctors this, doctor that. It is a presentation of power over this individual that is lying in bed.”*

While a few providers stated that they allowed caregivers to stay with patients with cognitive disabilities, participants shared that the extra steps taken to acquire approval were perceived as an additional burden by the providers. Participants also described the concerns for patients with disabilities who didn’t have a family advocate present. The lack of interaction between patients and their families caused significant emotional distress with families.

*“I would say my direct exposure would be more with the disabled population who, really, for the most part, was unable to advocate for themselves at all during COVID. And you don’t have your daughter, your son, your husband, your wife who normally is by your side every time you’re admitted to the hospital, who’s asking the questions that need to be asked, who’s questioning this medication decision or this test decision, or consenting to procedures, or who is the eyes and the ears.…And so, absolutely complaints about continuity of care, consistent communication with physicians, decision-making, "Why has he not gotten out of bed in two weeks?", "Why is his breathing like this today?" […] Of course, that brought a lot of complaints, and anxiety, and anger. […] And they weren’t allowed […] to even just come and stand outside the room; we had a no-visitor policy in our entire hospital whether you were COVID or not. […] I mean there was nothing unless we Zoomed with you.”*

There was also often mistrust or disagreement related to what choices for care were being made, because the family was not able to be present and see the care first-hand.

*““family members could not come into the hospital, they could not see their critically ill loved one except on a video screening. So there were a lot of underlying mistrust, it brought it like way up to the surface. I don’t think it was fundamentally different than what was there already, […I think it was usually a little bit more covered over. And that was very in your face. So I had […] multiple family members ask me when we do video updates […] if I was going to take away their loved one’s ventilator in order to allocate it to somebody else. And was I going to do that because they were poor or black or–I remember very vividly family members telling me–in New York City family members telling me that ‘don’t take away my loved one’s ventilator, we will find the money to pay you.’”*

Some participants highlighted policies regarding patient placement and how this contributed to inequitable care of BIPOC patients. Several participants discussed the VIP patient phenomenon where VIP patients, who were majority White, received better care where "*more time was spent at the bedside by the nurses*, *more consultants tend to be involved in their care*.*”* One participant noted racial inequity while explaining the differences in treatment between transformed temporary ICUs and conventional ICUs.

*“Those [transformed] units didn’t really have the same resources as, like, conventional ICUs like a nurse ratio of one to two patients. A respiratory therapist that’s always available, a pharmacist on hand, a nutritionist. And, kind of, patients that were sent to these transformed COVID-19 ICU units received decent care, but I would say probably not the same level of care as those in the conventional ICU. And I did notice that most of the patients in the transformed ICUs were not Caucasian; they were of different ethnicities and tend to be of lower, of socioeconomic status."*

#### Lack of work setting resources

Lack of resources included lack of adequate staff, PPE, interpreter services, funding, and collaboration across systems. Lack of staff affected the amount of care each provider had to provide, especially for nurses. Inadequate staffing had a direct impact on how providers perceived patients who need accommodations and resulted in inadvertent discriminatory treatment due to the “*logistics”* required to provide accommodations. As one participant noted:

*“And the interpreter was more used when you actually had time to use it, and people weren’t doing; it’s oftentimes a thing when somebody was having something immediate happening to them because they were just trying to get through that situation….And I just feel, like, because of COVID-19, nobody going in the room, you were, kind of, trying to do 20 things at the same time because you were the only person.”*

In addition to lack of staff and time, many hospital services also had limited availability of in-person interpreters before the pandemic and worsened with COVID-19. Staff mainly had access to interpreter devices, such as tablets or telephones, which were either limited in their availability, took time to use, and/or at times did not work due to the system being overwhelmed. In addition, the process for disinfecting the equipment between patients was also time intensive.

Work setting related issues were more commonly reported among providers in emergency medicine and critical care settings where resources and caseload were significantly affected by surges. Lack of collaboration across systems was also highlighted as contributing to decreased resources, staffing shortages, and systemic stress overall:

*“The organizational policies of New York are really different than in Washington State. And while the healthcare systems in Washington State don’t get along all the time,[…] they get along a lot better than the hospital systems in New York State and New York City do. And so I think you already had a relationship that was probably a little bit adversarial–competitive I should say. And it’s a lot more systems and I don’t think there was the same, there wasn’t the same coming together of a mutual aid.”*

#### Burnout and moral injury

Providers reported moral injury (a betrayal of one’s value and belief for not being able to help patients) and all elements of burnout including frustration, futility (people not getting better) and exhaustion [34, 35]. Moral injury was closely related to lack of resources and the busy environment as well as the emotional hardship related to COVID-19 specific care:

*"[COVID] has made it so that caregiving in general has just been a lot harder emotionally…*.*no one is really there for them at the end of their life*. *And it just feels like all of those things that we were taught in nursing school to do to provide family-centered care aren’t there*. *And it’s very hard*, *in general*, *to be watching people who are young dying*, *and to know that they’re dying alone*, *and you’re not actually able to help support their family*.

Burnout seemed to have a detrimental impact on care of BIPOC patients and patients with disabilities particularly those who required accommodations. Participants described when care became increasingly complex and required more time and resources but continued without *“any positive outcome”* it was *“hard to stay positive*.*”* This emotional distress and burnout often resulted in more explicit expression of bias.

*"It is hard to get out of [trash talking about patients] once you’ve been doing it for a while and that is reflective for many nurses. Initially as a coping mechanism and then as an expression of burnout…talking shit about your patients is universal. Not that that makes it okay, but yeah."*

Several providers showed evidence of burnout in the way they expressed anger and lack of compassion for unvaccinated patients (particularly before the omicron variant) who, as stated earlier, tended to be BIPOC patients in some areas.

*“If you didn’t get vaccinated and you get it [COVID-19] shame on you*. *That’s your big scarlet letter right there*. *This is not the politically correct answer*, *but I have no problem being judgy at this point*. *I’ve seen too many people die miserable*, *horrific deaths alone*. *Like if you didn’t get vaccinated and you get COVID sucks to be you doesn’t it*? *And that is definitely a reflection of burnout*."

## Discussion

This study explored provider observations of inequitable care provided to COVID-19 positive patients who are BIPOC and/or have disabilities and the drivers to inequitable care. While discriminatory care for BIPOC patients and patients with disabilities has previously been documented, our study confirms that the COVID-19 pandemic and related restrictions and policies have exacerbated such concerns. Findings suggest that BIPOC patients and patients with disabilities may be experiencing lower quality of healthcare related to COVID-19 in forms of decreased care, delayed care, and fewer options of care. This inequitable treatment was often driven by providers’ bias, organizational policies, lack of work setting resources, overwhelmed medical environment and provider burnout. In addition, the provider perspective in this study provides depth related to issues which may be unknown to patients and families, such as the nature of side-conversations, inadequate care, and fewer care options.

Lack of access to appropriate communication accommodations was a significant finding, both with respect to English and American Sign Language (ASL) interpreter needs and those with cognitive disabilities. Participants reported inconsistent use of interpreters or other communication technologies necessary for efficient and clear communication. This study also found that some providers deliberately chose to avoid or delay the care of people who need these accommodations due to the perceived extra burden. For patients with LEP, this lack of support was worsened primarily by isolation policies (i.e., interpreters or caregivers not allowed to enter room), restrictive visitor policies, lack of sufficient translation devices or burden around sanitizing devices, as well as providers’ perception that accommodations are not essential when busy. Most of these drivers were exacerbated with the busy environment which became more significant with the pandemic and surges. Providers’ attitudes played an even more significant role when it came to people with cognitive impairments, such as dementia or intellectual disabilities, as there were underlying assumptions that these individuals were not capable of understanding or making decisions in their care. Lack of compassionate care or avoidance were noted, which likely stems from providers’ bias and lack of competency in interacting and communicating with people with cognitive impairments [36, 37].

As found in other studies, the lack of accommodations was not recognized as discrimination by participants, raising both ethical and legal concerns [38, 39]. Participants noted a historical reliance on families as interpreters or on caregivers to assist with accommodations, without providing accommodations, and pointed to COVID-19 related restrictions and situations as problems, not recognizing the fact that accommodations must be legally provided at all times [40, 41]. Lack of communication accommodations is a significant health equity problem as it also has been associated with poorer health outcomes, including higher risk of readmission and increased incidence of adverse events [42–44]. This finding underscores the need to re-examine policies and processes related to communication accommodations for patients, including use of advanced technologies and expanding the workforce to include more language-concordant providers; further, provider training is needed to ensure that use of these accommodations is recognized as a human and legal right and as a norm, rather than the exception [38, 39, 45].

In addition to bias and assumptions related to language and communication barriers, the provider perspective supplied in this study highlighted concerning actions and attitudes directly connected to racism. Inequitable care such as decreased care or fewer options of care often occurred where there was intersectionality of socioeconomic status, cultural differences, a language spoken other than English. These actions were driven by providers’ stereotypes and assumptions related to race. In addition, local policies, such as how providers select patients, where patients were placed (ICU versus an auxiliary location), and flexibility in how care is scheduled further allowed for care to be influenced by provider bias. This aligns with previous research demonstrating a negative association between provider implicit bias against BIPOC populations and differences in clinical decision-making [7]. These realities only serve to increase the disproportionate risk for mortality from COVID-19 already faced by these populations [1, 22, 46].

Participants also frequently noted that racism was only present in side-talk and did not translate into care. However, providers’ attitudes and lack of compassion influence patients and their care experiences in negative ways. BIPOC patients often report feeling disvalued and not heard and having difficulties working with providers who do not understand their cultures and are not aware of power dynamics [6, 47, 48]. Because this study only included providers and the majority of our participants were white, it is possible that subtle discrimination or negative patient-provider interactions based on race (not LEP) were under-reported. Further examination of patient perspectives may be needed to better understand the impact of providers’ cultural incompetency on patients’ satisfaction and health outcomes.

The study also adds novel empirical evidence around how ableism impacts COVID-19 care for people with disabilities. Concerns have been raised that state triage protocols leave opportunities for providers to be discriminatory or biased to people with disabilities [49, 50], and media has documented anecdotal stories of patients with disabilities experiencing discrimination [51]. The findings of the current study confirm that providers’ ableist bias can result in negligent care practices; this was evident in participants’ descriptions of situations where providers skipped critical care due to their beliefs that people with disabilities have a lower quality of life and thus a life less worth living. In addition, some participants observed health care providers being held in check by fear of punishment and loss of license if they explicitly discriminated against BIPOC patients but not hesitating to openly share discriminatory treatments provided based on disability.

Lack of disability competency in the medical community is well documented; however, the ableist messages conveyed in COVID-19 public health guidelines (e.g., masking not required), healthcare policies (e.g., crisis standards of care), and speeches by government officials (e.g., expressing encouragement by the fact that death is higher among people with four or more chronic conditions) [52] could have worsened providers’ dismissal and devaluation of people with disabilities in care. As noted in the results, ableism can intersect with ageism and racism exponentially multiplying care inequities and subsequent risks when a patient is both BIPOC and has a disability. This is particularly problematic because life and death decisions are made based on providers’ evaluation when crisis standards of care are implemented during a public health crisis like the pandemic and, as noted above, current standards leave opportunity for discrimination to affect such decisions [5, 53, 54]. Organizations need to routinely monitor bias/discrimination-driven inequities and have systems in place to address these. Institutionalized policies (e.g., reporting of discrimination on the basis of disability or annual provider evaluations and performance improvement incorporating an appraisal of care for those with disabilities) need to be in place to hold providers accountable for inequitable care for patients with disabilities [55]. In addition, more attention needs to be paid to public messaging so COVID-19 is seen as a public health issue for all, not just a problem for people with disabilities or chronic conditions.

In addition to known drivers of inequitable care, such as provider bias and burnout, several drivers were unique to and/or exacerbated by the pandemic, including fear of transmission, lack of resources, organizational policies (e.g., visitor restrictions), and burnout. Understanding the individual and system level drivers underlying these inequities in care during the pandemic provides opportunity for preventive multi-level interventions in crisis planning and care. Interventions may include integrating enhanced provider training and policies around bias and discrimination as well as supportive services and systems. These efforts should be institutionalized so that during crisis situations like the COVID-19 pandemic, inequities are monitored and resolved in real time. Changes at the organizational level could include using resource allocation protocols that are equitably designed (e.g., remove age as a consideration, avoid assigning value based on a patient’s functional level) and provide less opportunity for bias and subjective judgement. While burnout does not excuse the discriminatory care observed by participants, it is an area that needs to be addressed, as highlighted by the recent National Academy of Medicine’s draft “National Plan for Health Workforce Well-being” [56]. Interventions implemented at the federal, state, organizational, and individual level that facilitate a more supportive work environment as well as healthier coping mechanisms among providers, address provider workload, provide adequate protection and support to providers (such as personal protective equipment) and invest in growing a more diverse healthcare workforce are an essential part of preventing discriminatory care [56, 57].

New policies could also be implemented which provide consistent exceptions for family members or another caregiver to be present in the hospital as an advocate to support communication or care decisions [3, 5]. However, this exception should not justify the lack of accommodations and supports from healthcare providers and the system. Hospitals need to re-examine their current services and resources for providing accommodations and establish a system that is sustainable even when the healthcare system and staff are overwhelmed. Voices of multiple and diverse stakeholders, especially patient advocates representing diverse identities, are critical in the process of developing these trainings and policies, with consistent use of an equity lens throughout [5, 58]. Overall, policies developed for a pandemic response need to be upstream in nature in order to avoid the inequities which have resulted from organizational decisions and organizational and staff bias.

The study has limitations. Recruitment occurred in four states in the United States only which potentially may not be representative of what is happening across all states or other countries. Although we reached saturation, we have a small sample size and the majority of our participants identified as white, females, and physicians, which limits the transferability of the study. In addition, the focus of the study was on perspectives of providers who worked in Federally Qualified Health Centers and inpatient settings at major hospitals. Future studies which include the perspectives of providers in long-term care settings could be beneficial, as could a focus on care of other marginalized groups and qualitative evaluation of interventions to improve equity in care. Finally, although we engaged in reflexivity, we acknowledge that our own biases and perspectives may have influenced the interpretation of data. Despite these limitations, the study offers critical and novel information which can inform policy decisions and interventions to ensure equitable care of patients who are BIPOC or who have disabilities.

## Conclusion

The study illustrates ways that BIPOC patients and patients with disabilities may experience lower quality of COVID-19 related care, compounding existing inequitable care for these populations. Drivers due to COVID-19, including fear, lack of needed resources, and COVID-19-related restrictions and policies added to known drivers, such as providers’ biases and burnout; all were exacerbated by the overwhelmed healthcare system. The findings underscore the importance of integrating multi-level preventive interventions as the pandemic continues and in preparation for future health crises. Voices of multiple and diverse stakeholders are critical in the process of developing these interventions.

## Supporting information

S1 Checklist COREQ (COnsolidated criteria for REporting Qualitative research) checklist. (PDF)

S1 Data Raw data used in developing major themes. (DOCX)

S1 Text Interview guide. (DOCX)

## Abbreviations

BIPOC: Black, Indigenous, and People of Color
LEP: Limited English Proficiency
