# Supplementary material for: Inequitable care delivery toward COVID-19 positive people of color and people with disabilities
Source: PLOS Glob Public Health. 2023 Apr 19;3(4):e0001499. doi: 10.1371/journal.pgph.0001499 (PMC10115306; doi:10.1371/journal.pgph.0001499)
Supplement: S1 Data — (DOCX) [file pgph.0001499.s002.docx]

Contents

[Discriminatory Treatment 2](#_Toc93406525)

[**1.** Decreased care: less care, less explanation of care, less access to care, less time at the bedside, less access to specialists 2](#_Toc93406526)

[**1.1.** Lack of accommodations: interpreter – English, ASL; resulting in less explanation, less interaction --- not getting information in their primary language, don't get the same level of information as someone who speaks English (also showing up in negligence) 3](#_Toc93406527)

[1.2. Lack of accommodation: caregiver support 5](#_Toc93406528)

[1.3. Discriminatory attitude/bias directly influencing behaviors 5](#_Toc93406529)

[1.4. System set up to be discriminatory (different quality of care) 6](#_Toc93406530)

[2. Delayed care (due to avoidance, system, personal attitude, being busy/added effort) 7](#_Toc93406531)

[3. Fewer options for care 7](#_Toc93406532)

[Drivers 8](#_Toc93406533)

[1. Negative Talk and Attitudes, Bias 8](#_Toc93406534)

[**1.** Bias (low SES, documentation status, race, vaccination status, pre-morbid conditions) - both lack of compassion (around situation) and irritated at what they require 8](#_Toc93406535)

[**2.** Describing frustration due to client's documentation status and SES, being burdened by them 8](#_Toc93406536)

[a. Undocumented immigrant 8](#_Toc93406537)

[b. SES status: 8](#_Toc93406538)

[c. Disability 9](#_Toc93406539)

[d. Vaccination status 9](#_Toc93406540)

[**3.** Not acknowledging different culture - Clashes with cultural approaches to care 9](#_Toc93406541)

[**4.** Stereotyping: Categorizing based on race 10](#_Toc93406542)

[**5.** Fear of transmission (earlier during the pandemic) 10](#_Toc93406543)

[**6.** COVID-19 Restrictions: system/policy level 11](#_Toc93406544)

[**7.** Lack of Resources (staff, interpreter services, funding, or collaboration across health systems**)** 11](#_Toc93406545)

[**8.** Burnout 11](#_Toc93406546)

[**9.** Health care provider – patient power dynamic 11](#_Toc93406547)

[Consequences 12](#_Toc93406548)

**Provider observations of Discrimination**

Stigma Practices can include stereotypes (i.e. beliefs about characteristics associated with the group and its members), prejudice (i.e. negative evaluation of the group and its members), stigmatizing behavior (i.e. exclusion from social events, avoidance behaviors, gossip), and discriminatory attitudes (i.e. belief that people with a specific health condition should not be allowed to participate fully in society).

# Discriminatory Treatment

delayed or decreased care, less explanation about care, and fewer options for care, often due to the additional needs these patient groups require based on language barriers, cultural differences, lower socioeconomic status, and physical or mental impairments.

## Decreased care: less care, less explanation of care, less access to care, less time at the bedside, less access to specialists

### Lack of accommodations: interpreter – English, ASL; resulting in less explanation, less interaction --- not getting information in their primary language, don't get the same level of information as someone who speaks English (also showing up in negligence)

- - "when there’s **not that direct transmission of information, there’s things that are lost, there’s things that are misinterpreted. And so I would say that it would impact their care.** Even something as simple as COVID-19, you're supposed to prone all the time whether you're independent, whether you're on a ventilator, **you're supposed to prone. And that message I feel like is often just brushed away because they’re like that’s not that crucial to them**."
  - "didn't understand because he didn't speak English…didn't impact his medical care, but it impacted him personally for sure…it did play a role in his care."
  - "nurses would have to have time in their unbelievably crazy, busy, stressful COVID day to dial a loved one and allow them to have a conversation via Zoom…if it was ESL…then you'd have to find [a computer designated for a language line] plus the computer for Zoom" "how frequently did that happen? My bet is not much"
  - Pre-COVID "using the interpreter pad more, so there's a lot more advocacy for people, and a lot more helping explain to them"
  - **"Latino population depended upon a staff member translating for them.** And you have to remember that not all Spanish is correct Spanish. It’s broken Spanish. So **the messages weren’t coming across clearly and you don’t really know what was coming across. […] A lot of people walked away not getting services, but were they offered it? We don’t know because we don’t know exactly how that translation was happening**."
  - I definitely feel like **people who are not English speakers get almost worse care** because we’re not as proactive about talking to them in their primary language. […] we lose, even in discharge instructions too. You know, when people are like, what to do with…**they don’t get the same level of in depth discharge instructions** because they’re not primary English speakers
  - "Spanish speaking and blind. And I felt **the whole thing…he was just lost and confused with what was happening half the time because whenever he was…having a lot of trouble, people weren't stopping to explain it very well to him.**"
  - Patient who was deaf, no sign language interpreter, writing everything out "things can be misconstrued and things were not necessarily explained to the fullest capability"
  - **"the folks with cognitive disability--and also English as a second language, I think they really got the shaft during the whole thing**, to be honest--but the folks of cognitive disability and English as a second language, I think, probably had the least amount of communication with physicians; and during the height of COVID, **the majority of our physicians weren't even going in the rooms, they were observing outside the room or making phone calls into the room with these folks who some of them, they couldn't even understand English;** or B, don't even understand the concept of like picking up a phone and talking it on it anymore because their dementia is so bad or their cognitive impairment is so bad. So, absolutely, the time that those patients were communicated with, and things were explained, and time was spent with them was limited."
  - "there have been times where it’s been people of color who don’t speak English who have been treated unfairly in my opinion. Doctors aren’t going in the room because of needing to preserve PPE but doctors are pretty important. I feel like they should have been going in the room. So **because they’re not going in the room, nothing is being conveyed to the patient besides what the nurse can say […] if it’s someone who does speak English [the doctor] can call them on the phone and have that conversation with them. They’re not taking the time to get a two way phone to translate with them** over the phone so they just rely on what the nurses say. Which, not bad, nurses are great, I don’t mean to say that. But, they’re super busy too."
  - "there’s always that language barrier. They had a harder time getting through the system when **there's a system that’s already set up to not automatically care for people who don’t speak English as their first language**. … It’s always harder when somebody speaks a different language. … They get kind of put back into the room with less information than you would have about a patient who spoke English as their first language. And that leads to kind of a barrier of care. And you have to go in COVID precautions plus you have to have an interpreter video in there. It’s like these two barriers that make it … it takes longer to see that patient and for them to get the attention that they might get if they didn’t have that language barrier. Then I think … for a healthcare provider, **there’s not a lot of empathy for people who don’t know how to navigate the healthcare system."**
  - **"**A language barrier is a huge thing. I mean, I’m an immigrant to this country myself, and I know how that felt to me when I had a language barrier as well personally. So I can empathize with both ends because I’ve been at both ends basically. **It does create difficulties, especially as the care gets more technical and more complicated, to bring the information to the patient, because you’re so sick and you’re supposed to listen to the doctor or the nurse and to understand complex issues.** And also then, you ask the person, well, what would you like to have done? How does he or she make that decision? I don’t know how that can even be fixed, to be honest, because it’s so dynamic, but we try our best. But **it is difficult to take care of patients who have a language barrier on any given day, but I think COVID just adds to that dynamic just with everything else that’s going on.** "

**Amplified with no-visitor policy: COVID-19 added layer to this in that (1) rooms were in isolation, (2) no-visitor policy - lack of family advocacy available, (3) higher rates of people of color being hospitalized with COVID-19

- - "We have a big Hispanic population that works on the farms out here, seasonal laborers and there’s been COVID among that population. **When they come in, you know, we’re limiting family. There’s no family….95% of the time their family translates for them.** And then if the interpreter machine is in a different room and you put it into the COVID room that machine's out of commission for however many hours they’re in the ER and then it has to be cleaned and so you tend to sort of like lean towards like hey, do you speak enough Spanish to assess this guy so I don’t have to pull the interpreter in here?"

### Lack of accommodation: caregiver support

- - - If have disability - needing someone to advocate for you (both caregiver and provider) "just this extra layer that needs special approval…of making sure the nurse and provider is ready to advocate for you in this."
    - Family of color - man with disability - wouldn't let mom stay initially, she was going to take him home…
    - "**the folks with cognitive disability**--and also English as a second language, **I think they really got the shaft during the whole thing**, to be honest--but the folks of cognitive disability and English as a second language, I think, probably had the least amount of communication with physicians; and during the height of COVID, **the majority of our physicians weren't even going in the rooms, they were observing outside the room or making phone calls into the room with these folks who some of them,** they couldn't even understand English; or **B, don't even understand the concept of like picking up a phone and talking it on it anymore because their dementia is so bad or their cognitive impairment is so bad.** So, absolutely, the time that those patients were communicated with, and things were explained, and time was spent with them was limited."

### Discriminatory attitude/bias directly influencing behaviors

#### Ableism: Disability – not worth living – resulted in less care

- - Those that were technology dependent "**[heard] staff arguing that they did not want to take care of those patients, and risk their own safety when they had, kind of, a poor outcome expected anyway.**"
  - "One specific patient was severity RDS, paralyzed, prone on very high vent settings, on pressors, certainly at risk of dying at any time, had multiple arrests, in fact. And **I heard multiple staff members say that they were paying more attention to other patients than that patient because they didn't believe her to be likely to survive. And she was no different than any other patient other than the fact that she had trisomy 21."**
  - Reporting patient perspective - "patients feel like there’s nobody taking care of them because they’re in an isolation room and it takes a long time for a nurse or a doctor or whoever to answer their call, and then even longer for someone to physically be in the room. For somebody with any sort of physical limitation where getting out of bed hooked up to oxygen is hard, right? And then there’s the dyspnea and all the symptomatic limitations. So I think it may -- just feeling like you were cared for less than optimal."
  - Patient with Downs Syndrome - "**They just didn't want to give him the time in his care that I felt like he needed and he deserved to make sure they were examining him properly**, make sure that they weren’t missing anything with him. This was someone that was on the ventilator for 30 days. They couldn't get him off the ventilator. […] it could have been **kind of a neglective care like I’m not going to really pay much attention, he doesn't really get it, he’s not paying attention to what I’m doing,** was kind of the overall feeling I got in rounds which I did not love."
  - "if someone comes in more chronically ill, or more disabled, the same efforts are not always put in"

#### Bias: Race related assumptions resulting less care

- - Assumptions about pain tolerance: "They [the Latinx community] have higher pain tolerances' is something I heard over and over again."
  - "healthcare…has shifted to a way of **devaluing a person in these small ways…so I feel like people of color with their symptoms…sometimes they're taken less seriously, there's less compassion**…they're kind of almost unseen and not really talked about…and if a person of color has an advocate…I feel like you see a little bit of change in behavior from a medical provider because there is that additional accountability."
  - Labeled a "difficult patient" - intersects with cultural differences
    - Families declining provider recommendations and following what the family wants "becomes an issue when the doctors and nurses are pushing for one thing and now it's a difficult family and a difficult patient…I do feel like their care changes when that happens"

### System set up to be discriminatory (different quality of care)

- - - Patients categorized as "VIP" - reported as more often being White - "more time was spent at the bedside by the nurses, **more consultants tend to be involved in their care**"
    - "we had to create, to transform non-ICU space into these COVID-19 units to take care of patients. And those units didn't really have the same resources as, like, conventional ICUs like a nurse ratio of one to two patients. A respiratory therapist that's always available, a pharmacist on hand, a nutritionist**. And, kind of, patients that were sent to these transformed COVID-19 ICU units received decent care, but I would say probably not the same level of care as those in the conventional ICU. And I did notice that most of the patients in the transformed ICUs were not Caucasian; they were of different ethnicities and tend to be of lower, of socioeconomic status**."
    - "being where there are many different healthcare systems there was very little load sharing between healthcare systems. And so some systems were profoundly overwhelmed, while resources existed nearby in other systems. And there was not a structure or anything in place to really look that comprehensively, so – and the systems experientially that I think were more overwhelmed were just proportionally medically underserved communities, including those in neighborhoods that were more severely affected as well as healthcare systems that served disproportionate medically underserved communities, which also correlates to high proportions of BIPOC patients."

## Delayed care (due to avoidance, system, personal attitude, being busy/added effort)

- - "And then over at the U, I think the main thing that happens with the care decision is just it takes an ED to notice people because in the ED we choose our patients. We like watch the board and then we pick a patient. And you watch those patients sit there, **nobody picks them for a while, the providers.**"
  - "system is already set up to not automatically care for people who don't speak English as their first language…[patients] get put back into the room with less information than…a patient who spoke English as their first language. And that leads to a barrier of care…**it takes longer to see that patient and for them to get the attention that the might get if they didn't have that language barrier**."
  - "People are often very thoughtful about when they're going to go into the room because they know it will be a big ordeal…because it's more difficult sometimes, it gets done at a later time."
  - Gaps in ancillary services - "**I think if they [ancillary service providers] know it's going to be something that's going to take a lot of time, they'll either do it really early or really late.** And if it gets done really late or if it gets scheduled really late, and **sometimes other things take some time, then that person gets missed for the day.**"
  - Delayed procedure due to status (undocumented)
  - Psych patient - in crisis - staff declining to care for them because of this
  - "Then there's like ‘emotional and aggressive difficult’ where people don't want to go in the room because they're going to get verbally abused by the family"

## Fewer options for care

- [race x ses] Options not being presented specifically to people of color: "I saw a lot of instances where options were not being presented to people of color. And they would fault those social economic factors as, like, rationale. But at the end of the day that is very unethical in my opinion."
- Telehealth (different access to technology - some participants connected this to disadvantaging BIPOC patients): “we had to do a lot of our care -- this is more outpatient care -- sort of through telehealth, I think that definitely did put the BIPOC patients at a disadvantage mostly because -- and I did experience this personally. There’s limitations in terms of access to technology. If you are visually impaired, can you log onto a computer or a phone to start a video visit with the physician? Then some people were not sort of -- did not have enough technology literacy to complete that process."

Delayed Care

In addition to receiving decreased care, participants also reported instances of BIPOC and people with disabilities experiencing delays in care as a result of discriminatory practices and perceptions of the extra needs these patient groups required due to language barriers, cultural differences, and physical or mental impairments. When discussing how language barriers affect delays in care, one participant stated:

*[the] system is already set up to not automatically care for people who don't speak*  *English as their first language…[patients] get put back into the room with less*  *information than…a patient who spoke English as their first language. And that leads to*  *a barrier of care…it takes longer to see that patient and for them to get the attention that*  *they might get if they didn't have that language barrier.*

In other instances, participants emphasized the conscious and active nature of delayed care. For instance, one participant said, “*People are often very thoughtful about when they're going to go into the room because they know it will be a big ordeal…because it's more difficult sometimes, it gets done at a later time.*" Another participant expounded upon the same phenomena of avoidance, with an example from the Emergency Department, stating, “*And then over at the U,...in the ED we choose our patients. We like watch the board and then we pick a patient. And you watch those patients sit there, nobody picks them for a while, the providers.*

Another person talked about intentionality and the delay of care in ancillary services that BIPOC patients and patients with disabilities received, saying:

*I think if they [ancillary service providers] know it's going to be something that's going to*  *take a lot of time, they'll either do it really early or really late. And if it gets done really*  *late or if it gets scheduled really late, and sometimes other things take some time, then*  *that person gets missed for the day.*

Fewer Options for Care

Often times, patients who were BIPOC or who had physical or mental disabilities were given fewer options to care. When discussing this occurrence among people of color, socioeconomic status was often conflated with race. As one provider stated:

*I saw a lot of instances where options were not being presented to people of color. And*  *they would fault those social economic factors as, like, rationale. But at the end of the*  *day that is very unethical in my opinion.*

Telehealth was a type of care that was frequently cited as a limited option for people of color and those with disabilities. Emphasizing the limitations of telehealth for these populations, one participant emphasized:

*We had to do a lot of our care -- this is more outpatient care -- sort of through telehealth, I think that definitely did put the BIPOC patients at a disadvantage mostly because -- and I did experience this personally. There’s limitations in terms of access to technology. If you are visually impaired, can you log onto a computer or a phone to start a video visit with the physician? Then some people were not sort of -- did not have enough technology literacy to complete that process.*

Drivers

- Health care providers’ bias, lack of resources at their practice location (e.g., staff, interpreter services, funding, or collaboration across health systems), fear of transmission, and burnout were mentioned as drivers for discriminatory treatment.
- Mostly COVID-19 specific except for provider’s bias; intersection of COVID-19 related restrictions x lack of resources

## Negative Talk and Attitudes, Bias

**Blaming Language, Labeling/generalizing, making assumptions based on race/ethnicity and/or disability**: more related to race than disability, manifests as side-conversations, blaming language/narrative, judgment, labeling, assumptions, "trash talking"

### Bias (low SES, documentation status, race, vaccination status, pre-morbid conditions) - both lack of compassion (around situation) and irritated at what they require

- - - "the care was provided, but it was more like discussions afterwards in nursing stations"

### Describing frustration due to client's documentation status and SES, being burdened by them

### Undocumented immigrant

- - - "eating up resources" "he's still here because he can't afford a $24 item"
    - "who doesn't want a free ride here of course, this is better than going home by himself where he has no help" "he's being waited on hand and foot, who wouldn't want that"
    - "this is America" related to staff justifying different options or treatment

1. SES status: Frustration at the care they had to provide connected to patient's ADLs, level of sanitation in combination with judgements related to their racial/ethnic background (i.e. Black patient with lice - "if her hair wasn't so ratty" "if her hair was just properly kept")
   - - Frustration from staff regarding inability to discharge due to lack of resources to get necessary supplies, accommodations, undocumented status: "people began being more frustrated and making comments…closer to the point of him being medically ready, but not be able to go anywhere because he was illegal, 'no one's going to take him'… 'no home health company's going to be providing care' and stuff like that…"

### Disability

- - - "if that person was not as obese as they are, that this would most likely not be this severe right now. Or it wouldn’t be so many other components that were failing and would not create additional problems, not just fighting the COVID disease."
    - "with Trisomy 21, Down Syndrome, those kids tend to be very floppy, some of them…you need a lot of hands."
    - "logistics" of caring for those with extra needs (i.e. obese)
    - "the amount of logistics it takes to care for that person is enormous."

### Vaccination status

- - - "I don't feel like there is a lot of compassion towards the unvaccinated anymore. I feel like that's definitely worn down…and in our community [that population] is primarily Hispanic."
    - "**had a patient who was African American** […] **Didn't believe COVID existed, didn't believe in the vaccine. He, I think was treated unfairly.** He kept saying this doesn’t exist, stop telling me I have COVID and **they would just yell at him basically you have COVID, you have to believe it. And he just refused to believe it. And so I do feel like that does lessen the compassion.** He ended up coding four times and unfortunately he did not make it. And so, I don’t know, I wasn’t there for the code and so I don’t know how his attitude was portrayed to the code but they talked about it multiple times like he’s **a non-compliant patient. He doesn’t listen, and he happened to be African American."**

### Not acknowledging different culture - Clashes with cultural approaches to care

- - - "in the Hispanic population … family is very important and taking care of your family is what they do. I’ll see patients who are elderly who live with their children. That is just something that culture seems to really do. **And so focusing on taking them home, being with me, I’m going to protect them, I’m going to take care of them is part of it. And then I think the other part of it is a language aspect. So when these patients are in the hospital, the family members are really protective of making sure that someone is translating for them into Spanish. And with COVID-19 they don’t have that. And so I think even more so they’re like oh my God they’ve been in the hospital for two weeks, no one’s been talking to them** in Spanish, I’m taking them home, they’re mine. I’m protecting them."
    - "Especially, right now, with family members not being in the room, they don't necessarily see how bad their family is doing. And so people, I think, would oftentimes get frustrated that the families weren't understanding that their loved one wasn't going to get better; or wanting to stop interventions when other people thought it wasn't working. I think that that's **oftentimes a clash that people have when their values don't always agree with what the patient's family values are.** And I think **right now, even more than in the past because they're not in their room, families aren't really seeing that their loved ones really aren't getting any better**…. Caregivers start to feel frustrated…'this is, there's going to be a horrible torture, there's gonna be PEG, trached, and in a LTACH soon is what's going to happen at the best?' So I think that's, kind of, where a lot of the differences I see is that people get frustrated, especially with the families not understanding or being on very opposite pages in terms of the care that they want; and what, oftentimes, we want or we we we think would be best for the patient.

### Stereotyping: Categorizing based on race

1. "Hispanic panic" related to how Hispanic populations express grief, fear, and pain more outwardly

- "to standard White American culture it looks like drama…they find it to be unpleasant"

1. Black community communicates with more gestures- viewed as "aggressive" "crazy" "angry"

- "people mistake that for aggression sometimes, but it's just how people relate to each other"…"when that's misinterpreted as aggression, people act a different way…it's not infrequently that I'm told 'this Mom is crazy' or 'they're so angry', and then I'd go and talk to them…and they'll be fine."
- "the largest issue was…the group of 'they'. 'they cohabitate" 'they aren't staying home'…just automatically grouping a person [from a different racial group] when they come in into 'they'"
- One participant described comments they had overheard regarding this jump to grouping certain populations together - "'Well, they must live with seven people'…'They're dirty and none of them are wearing their masks' "
- "Or just making comments on the fact that those [with COVID-19] had primarily Latin last names"

## Fear of transmission (earlier during the pandemic)

- 1. “we do have a translation service, but… they were afraid to pick up the phone to use that language translator system….A lot of people walked away not getting services, but were they offered it? We don’t know because we don’t know exactly how that translation (through staff) was happening or what was said during that translation”

## COVID-19 Restrictions: system/policy level

- 1. No-visitor policy
  2. Isolation
  3. Appointments via telehealth
  4. Related to lack of family member presence - as advocates, interpreters

## Lack of Resources (staff, interpreter services, funding, or collaboration across health systems**)**

- 1. Worse in ICU and ER
  2. "because of COVID-19, nobody going into the room, you were, kind of, trying to do 20 things at the same time because you were the only person"
  3. Getting interpreter "emergent" "acting quickly" "something immediate happening…just trying to get through that situation

## Burnout

"hard to get out of once you've been doing it for a while and that is reflective for many nurses. Initially as a coping mechanism and then as an expression of burnout…talking shit about your patients is universal. Not that that makes it okay, but yeah." **Question here - separate out need to vent/process versus unfair/unkind judgements and language about patients?**

- - "the heavier the care towards an individual and the more manpower it requires, the longer it takes, the longer it does without any positive outcome…people just get tired."

## Health care provider – patient power dynamic

- **"the patients don’t maybe say what they think to the providers and to the doctors and the health care professionals, what they tell their families. And then when you talk to the families, you found out questions that they do not bring to us**…It just makes me question how do they perceive us, **when we walk in in our gear and equipment**, and especially when the doctor visitations are with all these **doctor this, doctor that. It is a presentation of power over this individual that is lying in bed**. And we don’t know what that person truly feels in their heart, because there’s like barriers—language, religious, cultural, racial, economics, you name it. What if that person is, for example, a person that is undocumented. Maybe he fears the system."

**COVID-19 related restrictions x lack of resources**

- - " it’s come up a lot in the discussions around the crisis standards of care triage, algorithms around you know, what is accommodation, what should accommodation look like, … Or similarly should the triage be blinded to the presence of disabilities. the decisions about resource of a patient can be so time sensitive, it’s very hard to imagine operationally what a nimble, timely, fast moving and the accommodation process looks like but addresses – with legitimately addresses concern."
  - Compounding factor in that the health system doesn't like to take time for when things aren't going according to protocol and plan "we have to be really cognizant when there's something that doesn't go smooth to take a moment to say that's okay, we should address this.": Relates to issues of **compliance,** maybe WHY extra needs lend to negative attitudes (interrupts the usual order and system of things)

# Consequences

- 1. Delayed recognition of complications, difference response resulting in different outcomes for patient
     - Relating to fear around COVID - not going in - "led to a delayed recognition of a primary illness that was not COVID, but nobody was doing neuro checks on her." - described as Asian patient
     - Assessing without interpreter
       - if the interpreter machine is in a different room and you put it into the COVID room that machines out of commission for however many hours they’re in the ER and then it has to be cleaned and so you tend to sort of like lean towards like hey, do you speak enough Spanish to assess this guy so I don’t have to pull the interpreter in here. And so, […] I would say the care is not as good, not by intent but because of the language barrier, you know?"
  2. Negative experience for patient - Affecting patient's own mental status, understanding, ability to explain their needs and get their needs met, patients reporting "feeling like they get less care"

**Uncategorized**

- [non BIPOC PWD specific] "I was on a dedicated unit so I was an inpatient nurse and **the unit was almost entirely black and Hispanic patients, … and they felt they were getting inferior care from their physicians because, they thought it because they were COVID patients, you know like the docs didn’t want to come on the floor and the docs wouldn’t see them face-to-face** and nobody would come and talk to them, the only person they saw were nurses. And **my assumption is that that’s true**. That didn’t actually have anything to do with race that had to do with the fact that there was a fear of COVID exposure."
- " I have witnessed, yet again, people are getting less options than their white counterparts in the setting of, like, needing a heart transplant or needing a lung transplant. And even the staff that I'm working with now, "Well, this is America,"
- "the West Coast is where I have seen a very different level of nursing care. Nurses are still … strongly avoiding these patients' rooms. These patients are not getting turned, they're not getting their care. They're not getting medications on time. They're getting a very different level of care."
- [not necessarily “care in hospital”] "care decisions about different services that were available or not available. Or you know there were programs like there was an isolation place and I think the system wasn’t set up for that isolation place to work well for people who didn’t speak English. And there were boxes that could go out and I think it took longer to get those boxes to the families who were Spanish speaking because there’s that extra barrier of connecting to that part of the community. … it’s more like the wraparound services that were needed with COVID. Like isolate. I think that people who are not white ended up having less access to those systems."
