# Supplementary material for: Inequitable care delivery toward COVID-19 positive people of color and people with disabilities
Source: PLOS Glob Public Health. 2023 Apr 19;3(4):e0001499. doi: 10.1371/journal.pgph.0001499 (PMC10115306; doi:10.1371/journal.pgph.0001499)
Supplement: S1 Text — (DOCX) [file pgph.0001499.s003.docx]

**Interview Guide**

Thank you for agreeing to speak with us. This is a study on experiences of stigma towards COVID-19 patients who are Black, Indigenous, and People of Color (We will call this group BIPOC) and/or people with disabilities. We define stigma as the process by which certain human characteristics are labeled as socially undesirable and linked with negative stereotypes about a class of individuals, resulting in social bias and discrimination. We are interested in hearing more about your observations of COVID-19 stigma as it relates to BIPOC and people with disabilities.

Before getting started, we would like to review some important elements of your rights as a research subject.

- First, this is a research study, and your participation is voluntary.
- The study involves one 30-minute interview, with possible follow-up at a later date if we need to clarify any information you provided.
- Your answers to our questions are confidential. Identifiable information will not be shared or associated with data in any presentations or reports.
- Study results will be reported in presentations and in reports that may be distributed from our website, in journals, etc. We will alert you when study results are available to the public, unless you prefer not to be re-contacted.

To ensure we capture your responses accurately, we would like to record this interview on Zoom. If you agree, recordings will be stored electronically, password protected, and be identified only by a study ID number.

If you consent, we will proceed with the interview. Do you consent to take part in this study and have your interview recorded?

1. [IF CONSENT IS GIVEN, TURN ON RECORDING] I have begun recording; do you mind saying “I consent to being recorded” so we have that on file? Thank you.
2. [IF CONSENT IS NOT GIVEN] Thank you for your interest in participating. Unfortunately, we cannot proceed with the interview without your consent. Thank you for your time.

**Interview Questions**

Before we dive into the main questions, we wanted to gather some demographic information.

- What is your gender?
- What is your racial/ethnic background?
- Do you identify as someone with a disability?
- What is your current role in your organization?
- How long have you been in this role?
- What is your primary area of practice?

As you have seen in the interview guide we sent, there are three main topics we would like to discuss today: First we want to talk about triage protocols and how they are applied, then about your experiences or observations of COVID-19 care provided to BIPOC and people with disabilities. Finally, we want to spend some time talking about COVID-19 stigma.

All questions will be centered on COVID-19 patients who are BIPOC and people with disabilities as we are interested in learning specifically about their care experiences during the pandemic.

[Triage protocol]

Starting with patient triage, nationwide, there have been surges of COVID-19 cases, but each area and setting has been impacted in different ways. Have you needed to refer to the Crisis Standards of Care (CSC or triage protocol) or hospital triage protocols to make decisions on COVID-19 patient care? [If no, skip this section]

1. Do you think the protocols have been applied equitably for BIPOC patients and patients with disabilities? Can you tell me about any occasions you have observed or heard about where the protocol was not equitably applied to those patients?

[Unfair treatment of BIPOC patients and patients with disabilities]

Now we would like to talk about COVID-19 care for patients of color and/or patients with disabilities. We would like to learn about your observations and experiences in your workplace.

- 1. Can you tell me about any occasions you have observed or heard about where COVID-19 patients of color and/or patients with disabilities were treated unfairly? (e.g., care decisions made differently, different attitude based upon race, ethnicity, disability, and socioeconomic status)

[Probes]

- - Have you noticed any signs of either unconscious or overt bias (e.g., negative attitudes, behaviors, stereotypes) towards COVID-19 patients who are BIPOC and people with disabilities?
    - Were appropriate accommodations/resources provided to meet their needs (e.g., physical access to medical equipment for wheelchair users, accessible health information for people with intellectual disabilities, translation support)
    - Are there other ways you have seen clinical interactions change during the pandemic that might have especially impacted care for COVID-19 patients of color and/or patients with disabilities? For example, the restrictions on number of people who can accompany a patient for health care visit, wearing a mask, interacting via telehealth, etc.
  1. Are you aware of any situations where COVID-19 patients of color and/or patients with disabilities have expressed concerns about their quality of care/ discriminatory treatment by health care providers?

[Stigmatization of COVID-19]

Lastly, we have some questions about COVID-19 stigma. Some illnesses, such as HIV/AIDS convey stigma upon patients. As a reminder, we define stigma as labeling people socially undesirable and linking them with negative stereotypes, resulting in social bias and discrimination.

- 1. Do you think COVID-19 has become a stigmatizing illness? If yes, how so.
  - For whom do you think it is most stigmatizing?
    - Probe: How about people of color and people with disabilities?
  - Have you experienced stigma because you provide health care to COVID-19 patients? What has been your experience? How does that experience influence your interactions with COVID-19 patients?
  1. Do you have thoughts about how to reduce or eliminate stigma associated with COVID-19?

**Thank you for answering these questions. Is there anything else related to stigma and care for COVID-19 patients who are BIPOC or people with disabilities that we haven’t touched on today that feels important to bring into our conversation?**
